# Supplementary material for: Sirt6 reprograms myofibers to oxidative type through CREB-dependent Sox6 suppression
Source: Nat Commun. 2022 Apr 4;13:1808. doi: 10.1038/s41467-022-29472-5 (PMC8980083; doi:10.1038/s41467-022-29472-5)
Supplement: Supplementary file 1 — Supplementary Information [file 41467_2022_29472_MOESM1_ESM.pdf]

Sirt6 reprograms myofibers to oxidative type through CREB-dependent Sox6 suppression

Mi-Young Song[1,*], Chang Yeob Han[2,*], Young Jae Moon[1], Ju Hyung Lee[3], Eun Ju Bae[2,$], and Byung-Hyun Park[1,$]

[1]Department of Biochemistry and Molecular Biology, Chonbuk National University Medical School, Jeonju 54896, Republic of Korea

[2]College of Pharmacy, Chonbuk National University, Jeonju 54896, Republic of Korea

[3]Department of Preventive Medicine, Chonbuk National University Medical School, Jeonju 54896, Republic of Korea

Running title: Sirt6 is a molecular switch regulating myofiber composition

Contents

1. Supplementary table

2. Supplementary figures

1. Supplementary table

Supplementary Table 1. Sequences for primers (forward, FOR; reverse, REV)

Gene (qPCR)	Sequences for primers
Myh7	FOR: ACAAGCTGCAGCTGAAGGTG<br>REV: TCATTCAGGCCCTTGGCAC
Myh2	FOR: CCAGCTGCACCTTCTCGTTTGCCAG<br>REV: CATGGGGAAGATCTGGTCTTCTT
Tnni1	FOR: TGAAGCCAAATGCCTCCACAACAC<br>REV: ACACCTTGTGCTTAGAGCCCAGTA
Tnnc1	FOR: AGCTCATGAAGGACGGTGACAAGA<br>REV: AACCGTGCAAGACCAGCATCTACT
Tnnt1	FOR: AAGGGGAGCGTGTGGATTTTG<br>REV: TCCTCCTTTTTCCGCTGTTCA
Myh4	FOR: CCTGGAACAGACAGAGAGGAGCAGGAGAG<br>REV: GTGAGTTCCTTCACTCTGCGCTCGTGC
Myh1	FOR: TGCAACAGTTCTTCAACCAC<br>REV: GCCAGGTCCATCCCAAAGT
Tnnt3	FOR: AACTGGAGACTGACAAATTCGAGT<br>REV: GCTGTGCTTCTGGGTTTGGT
Tnnc2	FOR: CCATCATCGAGGAGGTGGAC<br>REV: CTTCCCCTTCGCATCCTCTT
Tnni2	FOR: GCACCTGAAGAGTGTGATGCT<br>REV: TCTCCTTCTCAGATTCTCGGC
Ppia	FOR: GCATACGGGTCCTGGCATCTTGTCC<br>REV: ATGGTGATCTTCTTGCTGGTCTTGC
Mb	FOR: CATGGTTGCACCGTGCTCACAG<br>REV: GAGCCCATGGCTCAGCCCTG
Sdhb	FOR: CAGAGTCGGCCTGCAGTTTC<br>REV: GGTCCCATCGGTAAATGGCA
Fndc5	FOR: TCCTCTTCATGTGGGCAGGT<br>REV: GGGCTCGTTGTCCTTGATGATA
Nrf1	FOR: GGAGCACTTACTGGAGTCC<br>REV: CTGTCCGATATCCTGGTGGT
Tfam	FOR: GCAAAGGATGATTCGGCTCAGGGAA<br>REV: CCGGATCGTTTCACACTTCGACGG
Mtco1	FOR: CTACTATTCGGAGCCTGAGC<br>REV: GCATGGGCAGTTACGATAAC
Mtco2	FOR: AACCATAGGGCACCAATGATAC<br>REV: GGATGGCATCAGTTTTAAGTCC
Mcad	FOR: GGTTTGGCTTTTGGACAATG<br>REV: TGACGTGTCCAATCTACCACA
Atp5o	FOR: TCTCGACAGGTTCGGAGCTT<br>REV: AGAGTACAGGGCGGTTGCATA
Cox5b	FOR: TTCAAGGTTACTTCGCGGAGT<br>REV: CGGGACTAGATTAGGGTCTTCC
Cycs	FOR: CCAAATCTCCACGGTCTGTTC

	REV: ATCAGGGTATCCTCTCCCCAG
Ndufs1	FOR: TGCAAATCCCTCGATTCTGTTAC<br>REV: GCTTTCTCAATCTCTACCAGGC
Ndufv2	FOR: GCAAGGAATTTGCATAAGACAGC<br>REV: TAGCCATCCATTCTGCCTTTG
Nr4a3	FOR: TAGCCATCCATTCTGCCTTTG<br>REV: TGGTGTATTCCGAGCCATAAGT
Ppargc1a	FOR: ACGAGGCCAGTCCTTCCTCC<br>REV: AGCTCTGAGCAGGGACGTCT
Nr4a1	FOR: TGATGTTCCCGCCTTTGC<br>REV: CAATGCGATTCTGCAGCTCTT
Nr4a2	FOR: TGTCGTAATTCAGCGAAGGA<br>REV: TGAATGAAGAGAGCGGACAA
Sik1	FOR: CAGGTGCTAGGGATCATGCAG<br>REV: GGAGGTAGTAAATGGCGGCAA
Atf3	FOR: GAGGATTTTGCTAACCTGACACC<br>REV: TTGACGGTAACTGACTCCAGC
Creb1	FOR: AGCAGCTCATGCAACATCATC<br>REV: AGTCCTTACAGGAAGACTGAACT
Sox6	FOR: AATGCACAACAAACCTCACTCT<br>REV: AGGTAGACGTATTTCGGAAGGA
Cbx1	FOR: GGTGGAAAAAGTTCTTGATCGGC<br>REV: GCTCCCAAGTGTTGTCCTCA
Sp3	FOR: TGTCCCAACTGTAAAGAAGGTGG<br>REV: CTCCAGAATGCCAACGCAGATG
Purb	FOR: GATGTGAAGCAGAACGCCAAGG<br>REV: GCGTAGTGTTCTATGAAGTCGCC
Gapdh	FOR: GGCATGGACTGTGGTCATGA<br>REV: TTCACCACCATGGAGAAGGC
16S rRNA	FOR: AAATTTCGGTTGGGGTGACCT<br>REV: CCATTGGGATGTCCTGATCCA

Gene (Chip)	Sequences for primers
Creb1 Enhancer1	FOR: ACAGGTTGAGGGAGCAGAGA<br>REV: TTGGGTGGAAAGATGTGTCA
Creb1 Enhancer2	FOR: TGGTGCTAAAGCCAGTGTTG<br>REV: CATGCCTTCTCCACCAGTCT
Creb1 Enhancer3	FOR: AGGTGAAGAACTGGCGGTTA<br>REV: AAACAGATCCCCACCCTACC
Creb1 Promoter1	FOR: GGCGTCTCCAACCTTCCA<br>REV: GGGGACTTGCTGGAAACC
Creb1 Promoter2	FOR: GAAGCGGAGTGTTGGTGAGT<br>REV: GGTACAAGCTCCTCCGTCAC
Myh7 promoter	FOR: GCTCCAGCCCCTTTATATCC<br>REV: CAGACCTTTCATGGGCAAAC
Sox6 promoter	FOR: CAAGCCCAAGGTTTCGAGG<br>REV: GAAGCCATTCTCCATCATTGTT

Gene (siRNA)	Sequences for primers
Creb1 #1	Sense: CAGCUUUAACUCUGAUGUU<br>Antisense: AACAUCAGAGUUAAAGCUG

Creb1 #2	Sense: CUGUACAUAUGCUACUGAU<br>Antisense: AUCAGUAGCAUAUGUACAG
Creb1 #3	Sense: GAGUGUGUGCUAUGGUACA<br>Antisense: UGUACCAUAGCACACACUC
siSirt6	Sense: GUCUCACACACUCCACACA<br>Antisense: UGUGUGGAGUGUGUGAGAC

2. Supplementary figures

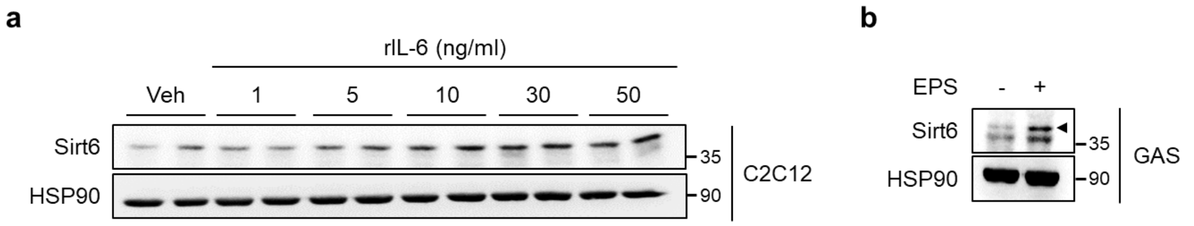

Supplementary Figure 1. Induction of Sirt6 by IL-6 treatment or electrical stimulation in C2C12 cells and gastrocnemius muscle. a Expression of Sirt6 was measured in C2C12 myotubes treated with recombinant IL-6 at indicated concentrations for 24 h. **b** Expression of Sirt6 was compared in gastrocnemius (GAS) muscle of mice with or without electrical pulse stimulation (EPS). All blot images are representative of at least three independent experiments.

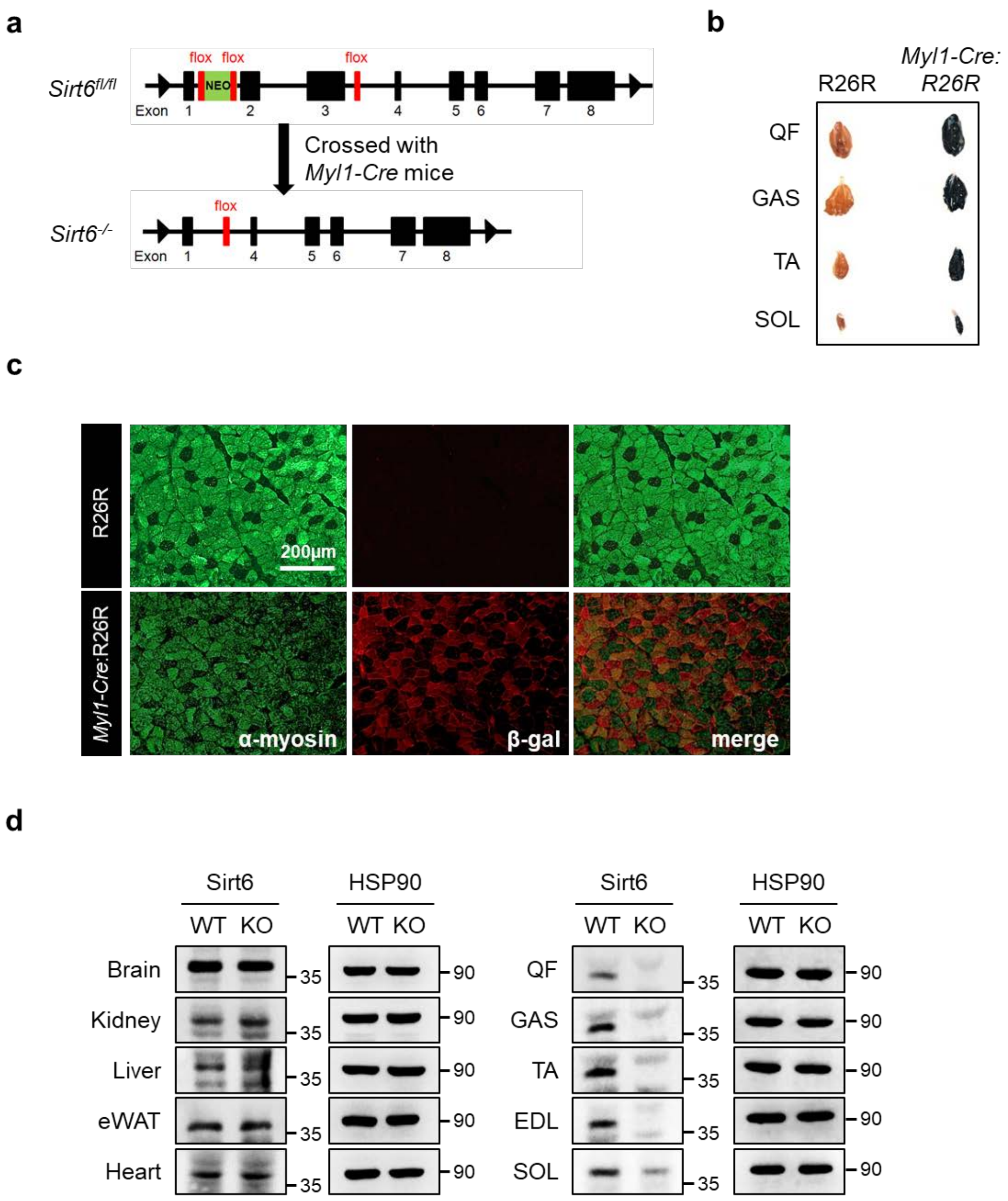

Supplementary Figure 2. Generation of skeletal muscle-specific *Sirt6* KO mice. a Schematic diagram illustrating skeletal muscle-specific ablation of *Sirt6* using *Myl1-Cre*. **b** Whole mount X-gal staining of hindlimb muscle tissues from three-month-old *Myl1-Cre:R26R* mouse after overnight incubation in X-gal staining solution. **c** Immunofluorescence staining for β-gal and α-myosin was performed on serial sections of GAS muscle. Bar=200 µm. **d** Western blot confirmation of Sirt6 ablation in *Myl1-Cre:Sirt6*$^{flox/flox}$ mice. All images are representative of at least three independent experiments. eWAT, epididymal white adipose tissue; QF, quadriceps femoris; GAS, gastrocnemius; TA, tibialis anterior; EDL, extensor digitorum longus; SOL, soleus

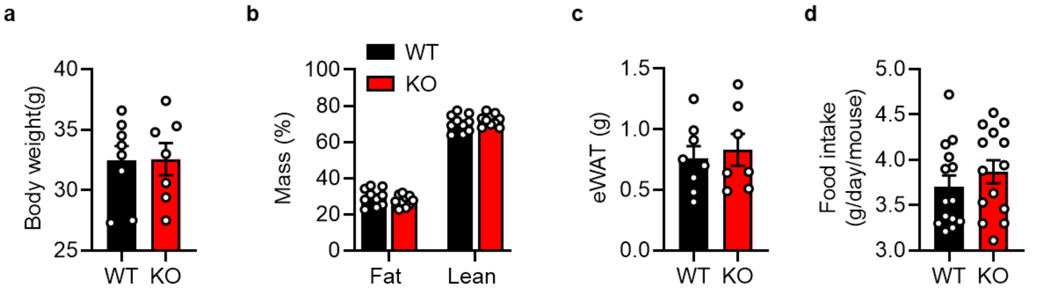

Supplementary Figure 3. Basic metabolic parameters of *Sirt6* KO mice. Body weight (**a**, n=8 for WT, n=7 for KO), fat and lean mass (**b**, n=10), epididymal white adipose tissue (eWAT) weight (**c**, n=8 for WT, n=7 for KO), and food intake (**d**, n=8 for WT, n=7 for KO) were compared in 20-week-old male WT and skeletal muscle specific *Sirt6* KO mice. Values are mean ± SEM. Data are representative of at least three independent experiments. Unpaired two-tailed *t*-test between two groups was conducted for statistical analyses. Source data are provided as a Source Data file.

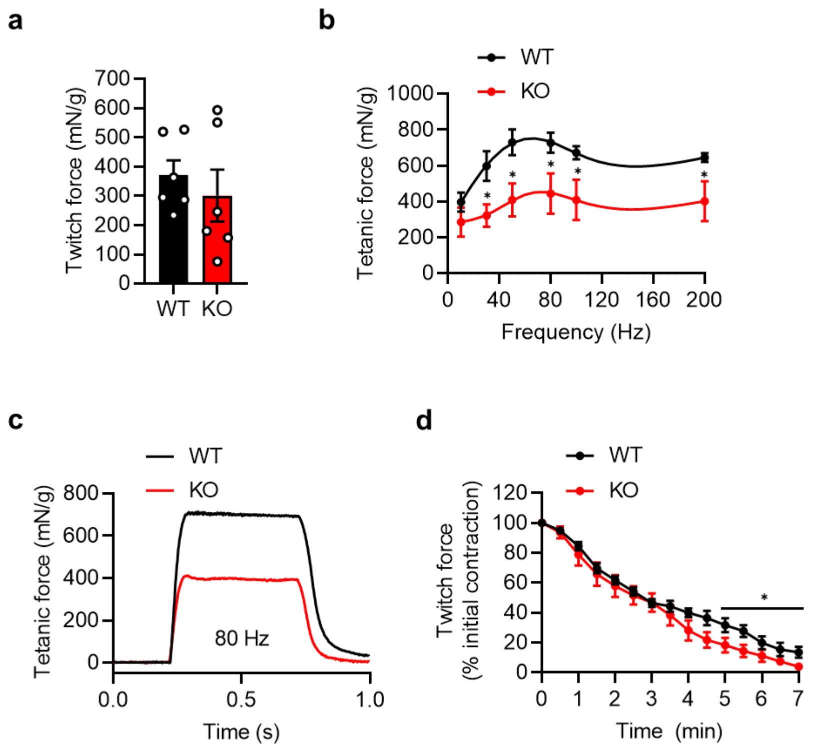

Supplementary Figure 4. Impaired force production and increased muscle fatigue in gastrocnemius muscles from *Sirt6* KO mice. a Isometric force and fatigue measurements were done from isolated mouse gastrocnemius muscles of WT and *Sirt6* KO mice (n=6). After mounting gastrocnemius muscles on a force transducer, the twitch force was measured by electrically stimulating the muscle with a single electrical pulse (100 V for 1 ms). **b, c** The tetanic force–frequency relationships were determined by triggering contraction using incremental stimulation frequencies (1 ms pulses at 10-200 Hz for 500 ms at 100 V, n=6). **d** Fatigue index was measured at 1 Hz and 100 V by repeated stimuli for 7 min and expressed as a percentage of the initial contractile force (n=5). Values are mean ± SEM. Data are representative of at least three independent experiments. Unpaired two-tailed *t*-test between two groups was conducted for statistical analyses (**b**, **d**). *, $p<0.05$. Source data are provided as a Source Data file.

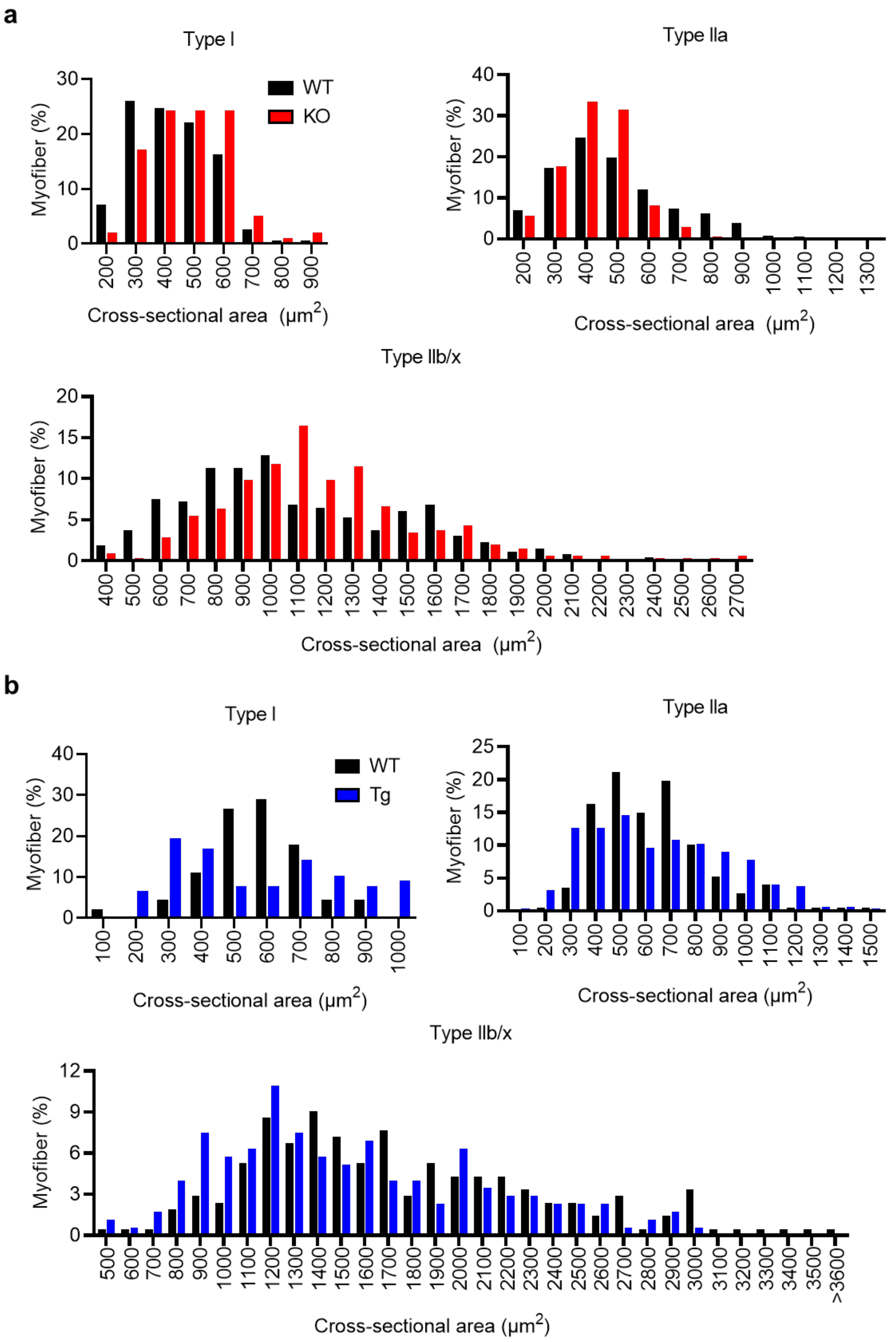

Supplementary Figure 5. Myofiber size distribution histograms in gastrocnemius muscles of *Sirt6* KO and Tg mice at basal conditon. a, b On the basis of expression of MyHC-positive myofibers in Figure 2b (**a**, n=6) and Figure 2f (**b**, n=4 for WT, n=6 for Tg), the cross-sectional area of each type was determined. Data are representative of at least three independent experiments.

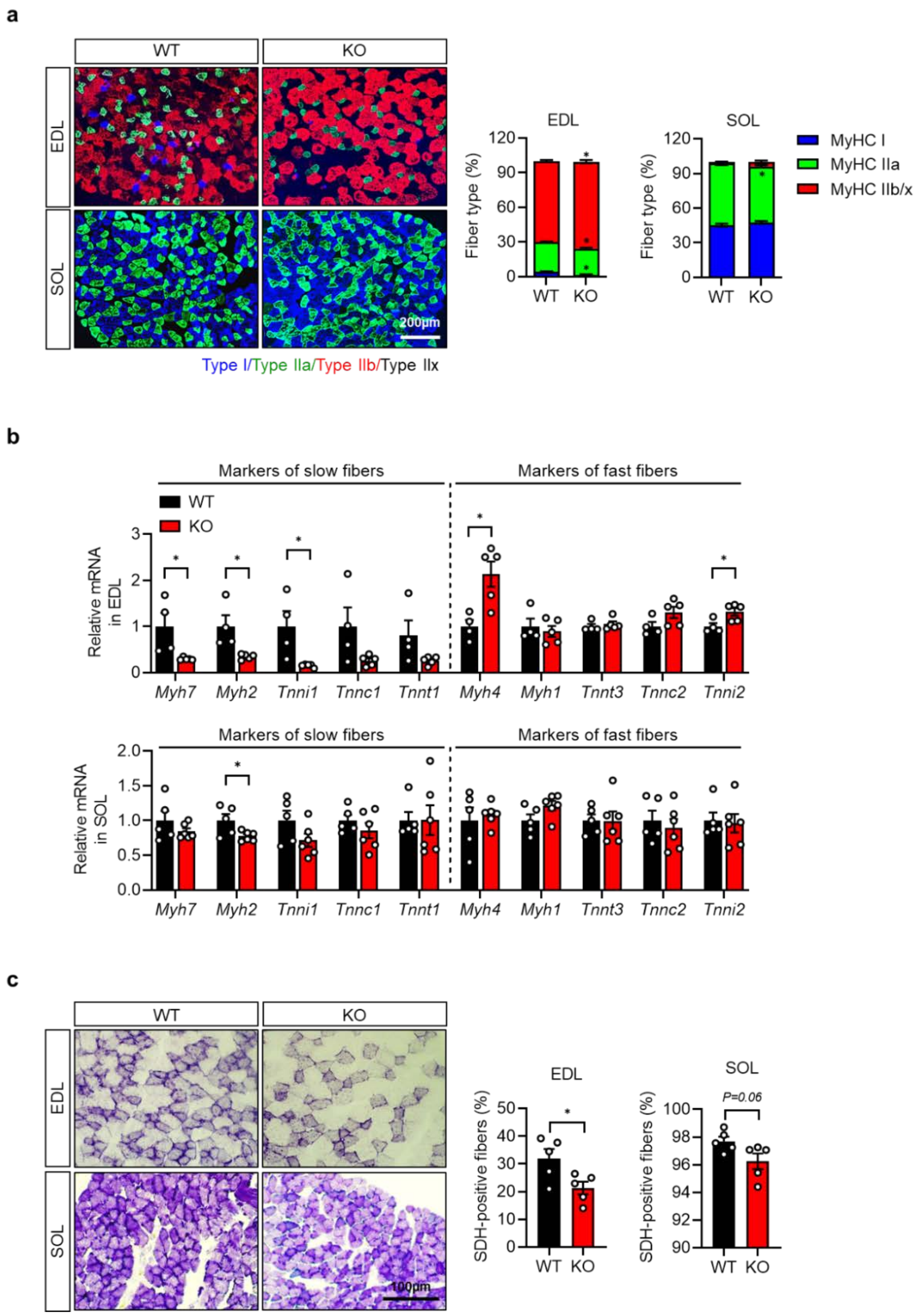

Supplementary Figure 6. Decrease in oxidative fiber density in the extensor digitorum longus (EDL) and soleus muscles (SOL) of *Sirt6* KO mice at basal condition. a Representative immunofluorescence staining for MyHC-I, MyHC-IIa, and MyHC-IIb/x. Composition of each myofiber was quantified (n=5). Bar=200 μm. **b** Expression of markers of slow and fast fibers was compared by qPCR (n=4-5 for WT, n=5-6 for KO). **c** Representative succinate dehydrogenase (SDH) staining and quantification of SDH-positive fibers (n=5). Bar=100 μm. Values are mean ± SEM. Data are representative of three independent experiments. Unpaired two-tailed *t*-test between two groups was conducted for statistical analyses (**a**-**c**). *, p<0.05. Source data are provided as a Source Data file.

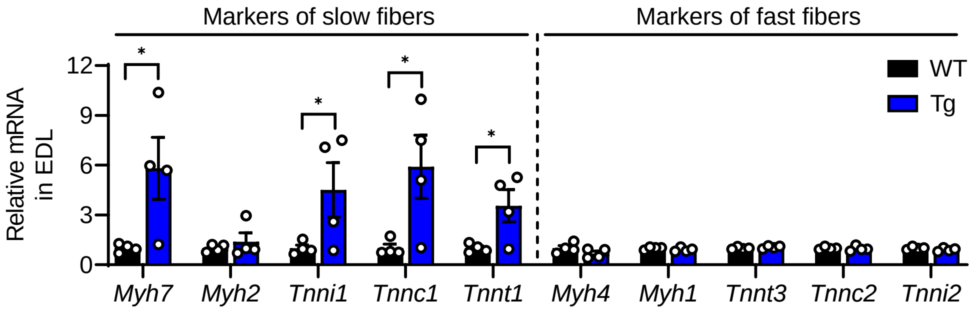

Supplementary Figure 7. Selective increase in oxidative fiber genes in extensor digitorum longus (EDL) muscle of *Sirt6* Tg mice at basal condition. Expression of markers of slow and fast fibers was compared by qPCR (n=4). Values are mean ± SEM. Data are representative of at least three independent experiments. Unpaired two-tailed *t*-test between two groups was conducted for statistical analyses. *, $p<0.05$. Source data are provided as a Source Data file.

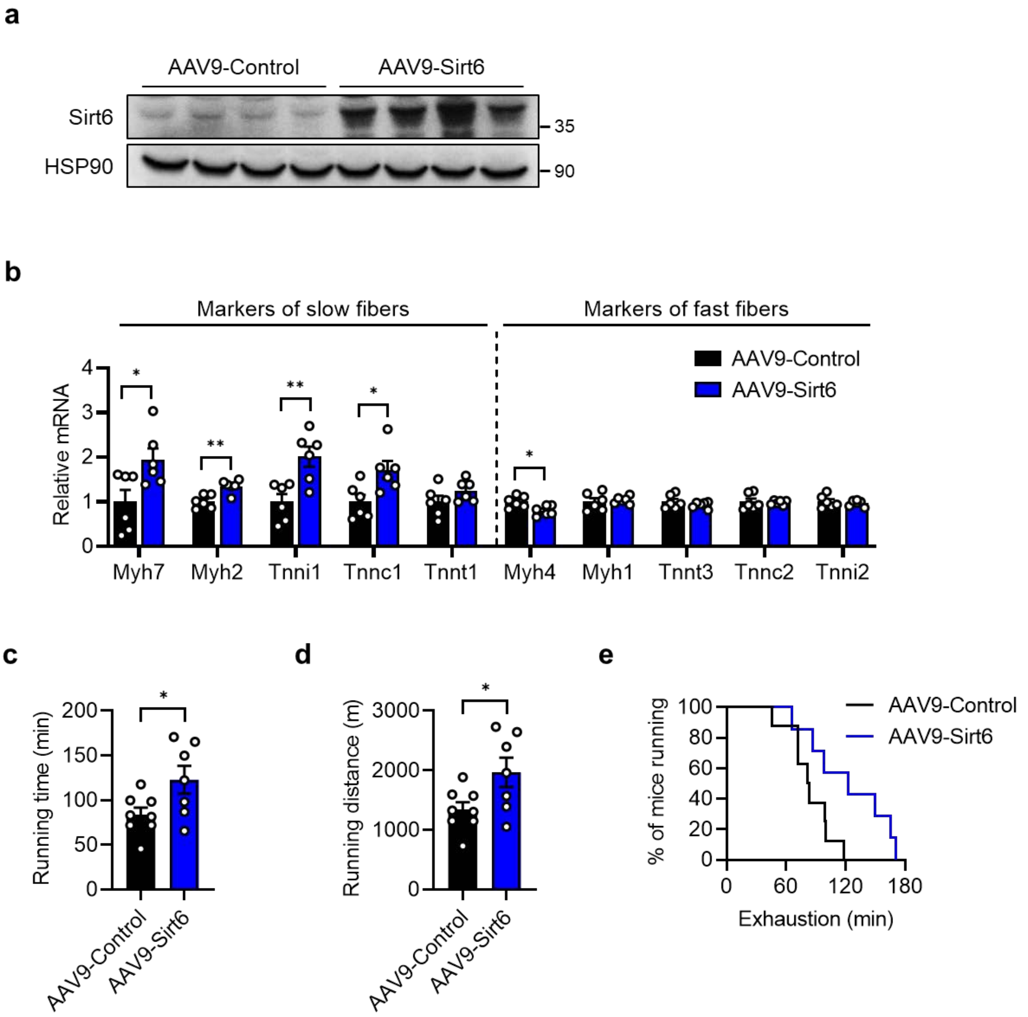

Supplementary Figure 8. Increase in oxidative fiber composition and exercise performance by muscular overexpression of Sirt6. a Overexpression of Sirt6 was verified by Western blotting in gastrocnemius muscles of mice injected intramuscularly with control or AAV9-Sirt6. For muscular overexpression of Sirt6, control or AAV9-Sirt6 was intramuscularly injected to mice and after 2 weeks, mice were subjected to treadmill running exercise for 4 weeks. **b** Expression of markers of slow and fast fibers was compared by qPCR (n=6). **c-e** Average running time, distance, and time to exhaustion after four weeks of treadmill exercise training (n=8 for AAV9-Control, n=7 for AAV9-Sirt6). Values are mean ± SEM. All Western blot images and data are representative of at least three independent experiments. Unpaired two-tailed *t*-test between two groups was conducted for statistical analyses (**b-d**). *, $p<0.05$ and **, $p<0.01$. Source data are provided as a Source Data file.

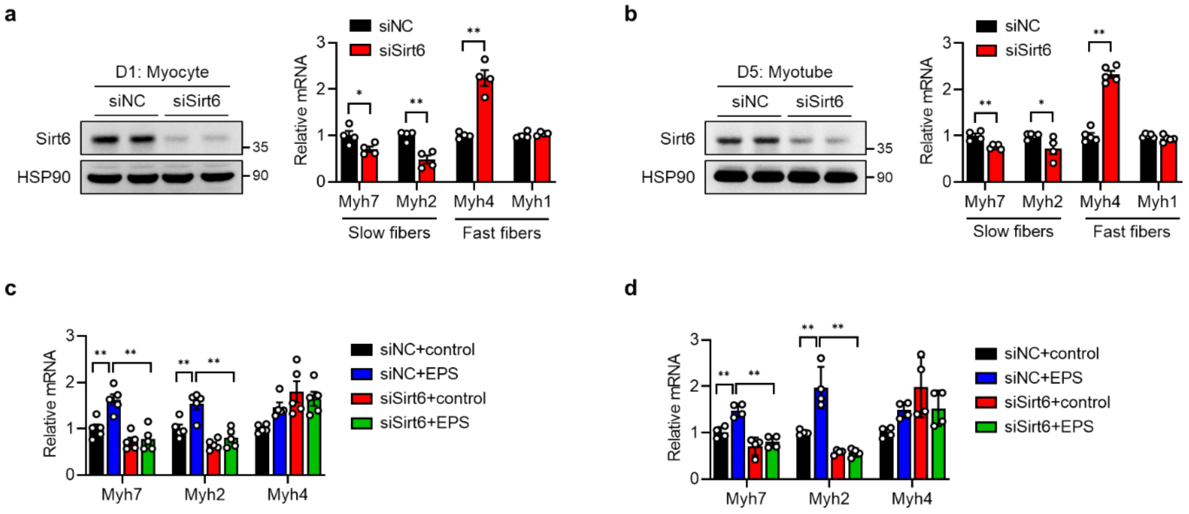

Supplementary Figure 9. Effect of Sirt6 silencing on myofiber specific gene expressions in different stages of myogenesis. a, **b** Slow and fast fiber gene expressions were examined by qPCR in C2C12 myotubes transfected with negative control (NC) or Sirt6 siRNA after differentiation (at day 1, **a** (n=4) or at day 5, **b** (n=4-5). **c**, **d** The experiments were done as in panel **(a)** and **(b)** in combination with electrical stimulation (1 ms pulses at 1 Hz for 6 h at 10 V) (n=5 for **c**, n=4 for **d**). Values are mean ± SEM. All Western blot images and data are representative of at least three independent experiments. Unpaired two-tailed *t*-test between two groups (**a, b**) and one-way ANOVA followed by Bonferroni's *post hoc* analysis (**c, d**) were conducted for statistical analyses. *, p<0.05 and **, p<0.01. Source data are provided as a Source Data file.

a

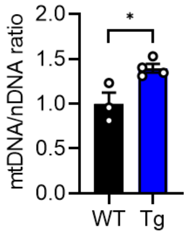

b

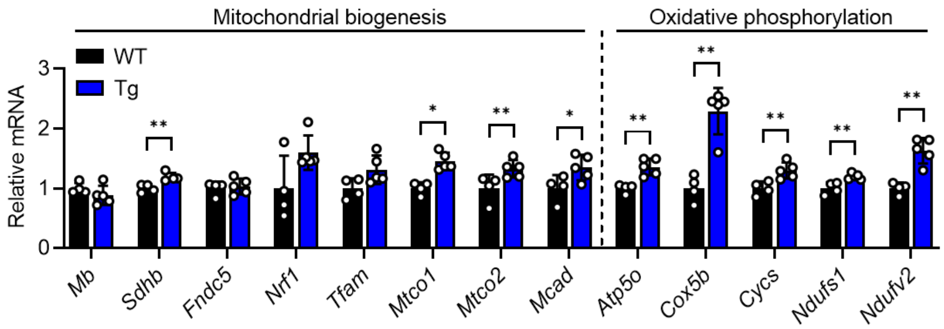

Supplementary Figure 10. Increase in mitochondrial biogenesis in gastrocnemius muscle of *Sirt6* Tg mice at basal condition. a Mitochondrial DNA (mtDNA) was quantified by qPCR using nuclear DNA (nDNA) as a standard (n=3 for WT, n=4 for Tg). **b** Expression of markers of mitochondrial biogenesis and oxidative phosphorylation was compared by qPCR (n=4 for WT, n=5 for Tg). Values are mean ± SEM. Data are representative of at least three independent experiments. Unpaired two-tailed *t*-test between two groups was conducted for statistical analyses (**a**, **b**). *, p<0.05 and **, p<0.01. Source data are provided as a Source Data file.

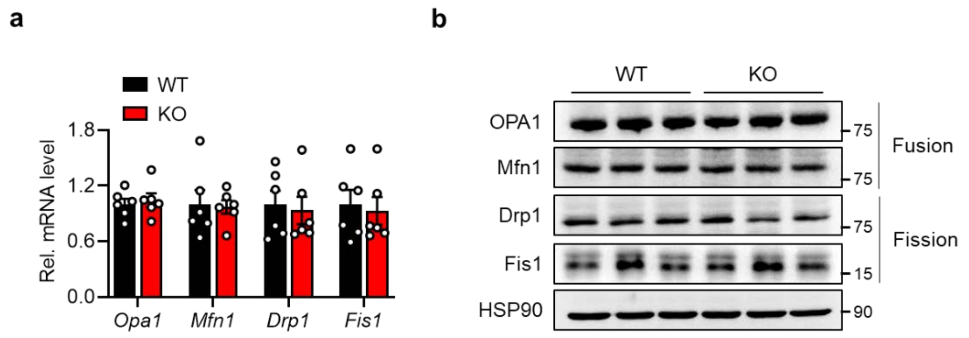

Supplementary Figure 11. No effect of Sirt6 deficiency on mitochondrial fusion-fission gene expressions at basal condition. a, **b** The mRNA and protein levels of genes involved in mitochondrial dynamics were examined in gastrocnemius muscles of WT and *Sirt6* KO mice (n=6 for **a**). Values are mean ± SEM. All Western blot images and data are representative of at least three independent experiments. Unpaired two-tailed *t*-test between two groups was conducted for statistical analyses. Source data are provided as a Source Data file.

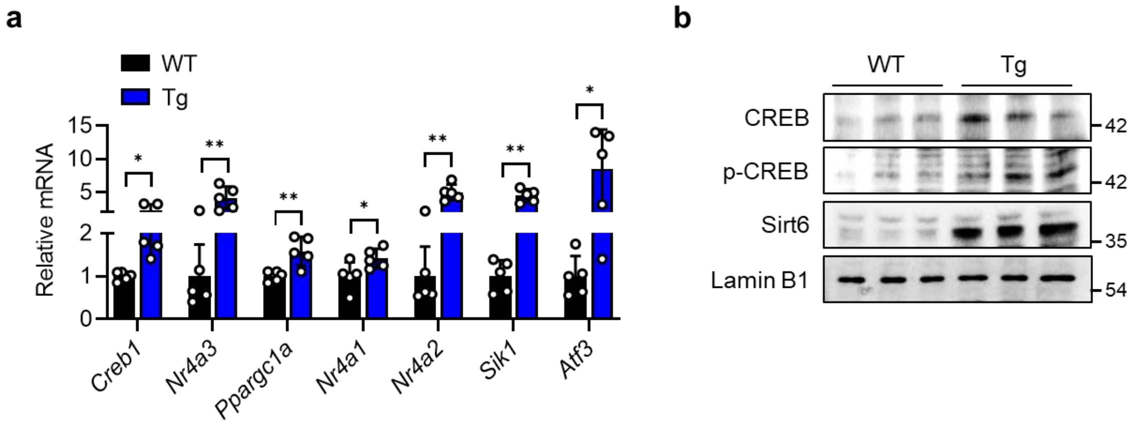

Supplementary Figure 12. Increased CREB pathway in *Sirt6* Tg mice at basal condition. **a**, **b** mRNA of *Creb1* and its downstream genes (**a**) and CREB protein levels (**b**) in WT and *Sirt6* Tg mice were analyzed by qPCR and Western blotting, respectively (n=5 for **a**). Values are mean ± SEM. All Western blot images and data are representative of at least three independent experiments. Unpaired two-tailed *t*-test between two groups was conducted for statistical analyses (**a**). *, p<0.05 and **, p<0.01. Source data are provided as a Source Data file.

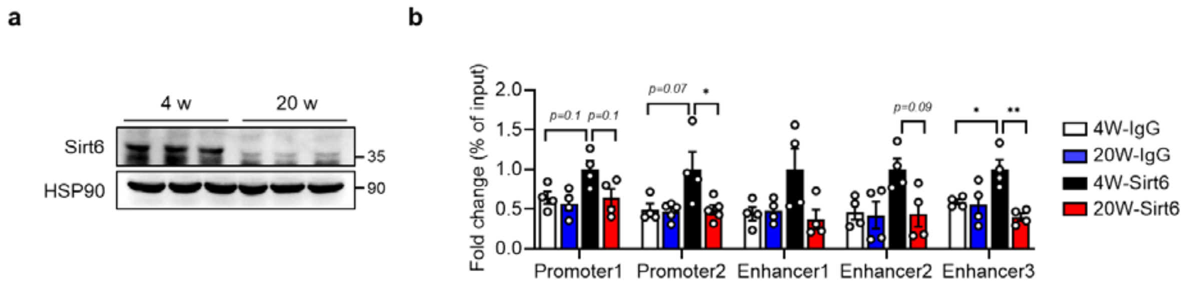

Supplementary Figure 13. Sirt6 protein level and its association on *Creb1* promoters and enhances in muscles of 4- and 20-week-old mice at basal condition. a Sirt6 protein level was examined in gastrocnemius muscle of 4- and 20-week old mice. **b** Occupancy of Sirt6 on *Creb1* promoters or enhancers was evaluated by ChIP-qPCR assay in mice muscle in (**a**) (n=4 for **b**). Values are mean ± SEM. All Western blot images and data are representative of at least three independent experiments. One-way ANOVA followed by Bonferroni's *post hoc* analysis was conducted for statistical analyses (**b**). *, $p<0.05$ and **, $p<0.01$. Source data are provided as a Source Data file.

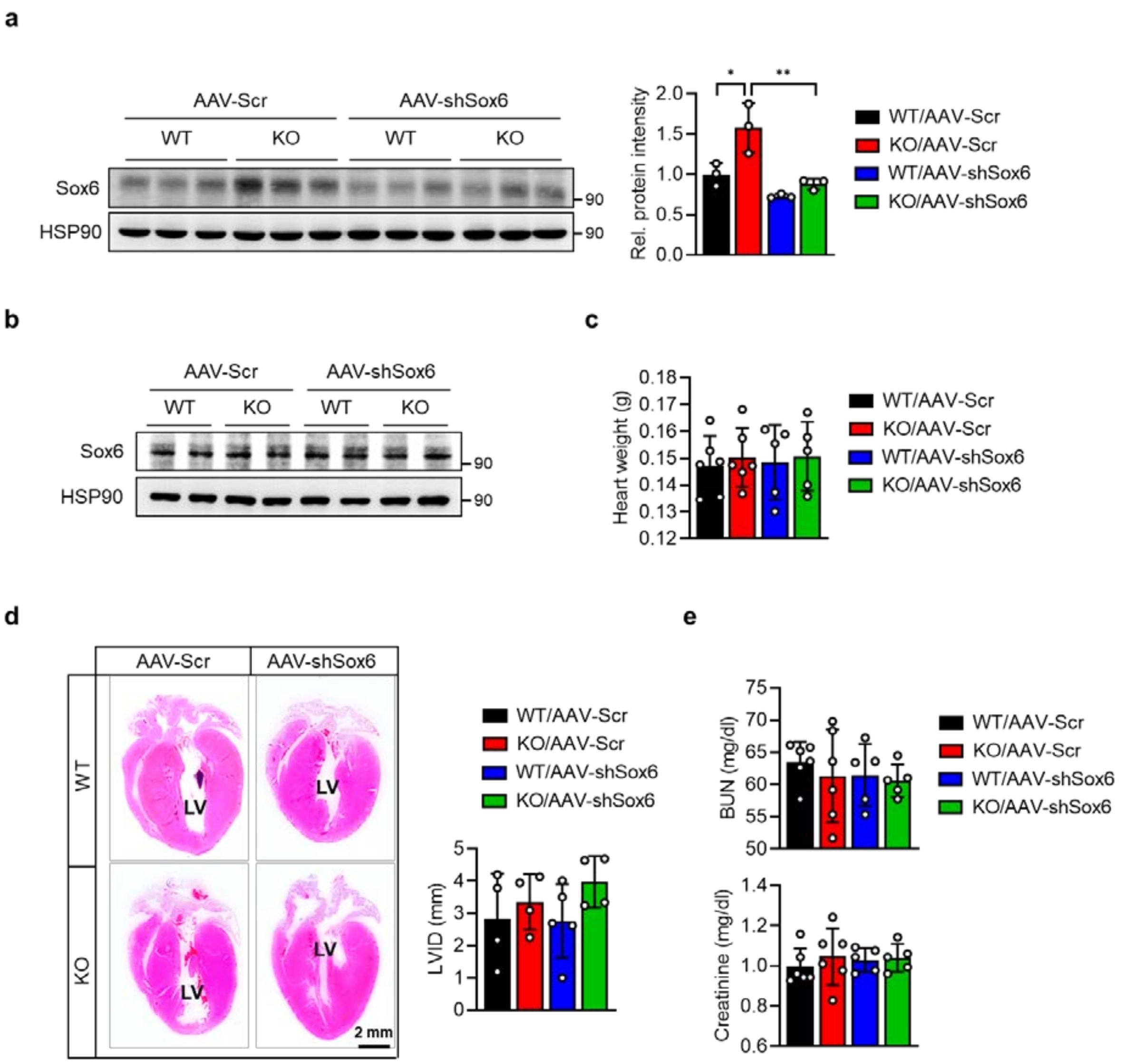

Supplementary Figure 14. Muscle specific knockdown of Sox6 with no effect on heart or renal function by AAV9-shSox6 delivery to mice. a, **b** Protein expression of Sox6 was examined in gastrocnemius muscle (**a**, n=3) and heart (**b**) of WT and *Sirt6* KO mice injected with AAV9-Scr or AAV9-shSox6. **c**, **d** The wet weight (**c**, n=6 for WT/AAV-Scr and KO/AAV-Scr, n=5 for WT/AAV-shSox6 and KO/AAV-shSox6) and H&E staining (**d**, n=5 for WT/AAV-shSox6, n=4 for other groups) of heart. Bar=2 mm. **e** Plasma levels of blood urea nitrogen (BUN) and creatinine (n=6 for WT/AAV-Scr and KO/AAV-Scr, n=5 for WT/AAV-shSox6 and KO/AAV-shSox6). Values are mean ± SEM. All images and data are representative of at least three independent experiments. One-way ANOVA followed by Bonferroni's *post hoc* analysis was conducted for statistical analyses (**a**). *, p<0.05 and **, p<0.01. Source data are provided as a Source Data file.

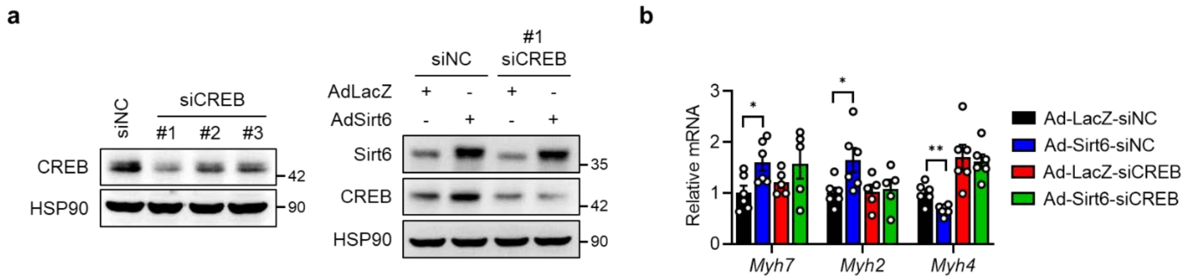

Supplementary Figure 15. CREB silencing reversed Sirt6 mediated expression of slow fiber genes. a Protein expression of the indicated genes was examined in C2C12 myotubes transfected with siRNA targeting negative control (NC) or CREB, which was followed by infection with Ad-LacZ or Ad-Sirt6. **b** The indicated genes were measured by qPCR (n=5 for Ad-LacZ-siCREB, n=6 for other groups). Values are mean ± SEM. All Western blot images and data are representative of at least three independent experiments. One-way ANOVA followed by Bonferroni's *post hoc* analysis was conducted for statistical analyses (**b**). *, $p<0.05$ and **, $p<0.01$. Source data are provided as a Source Data file.

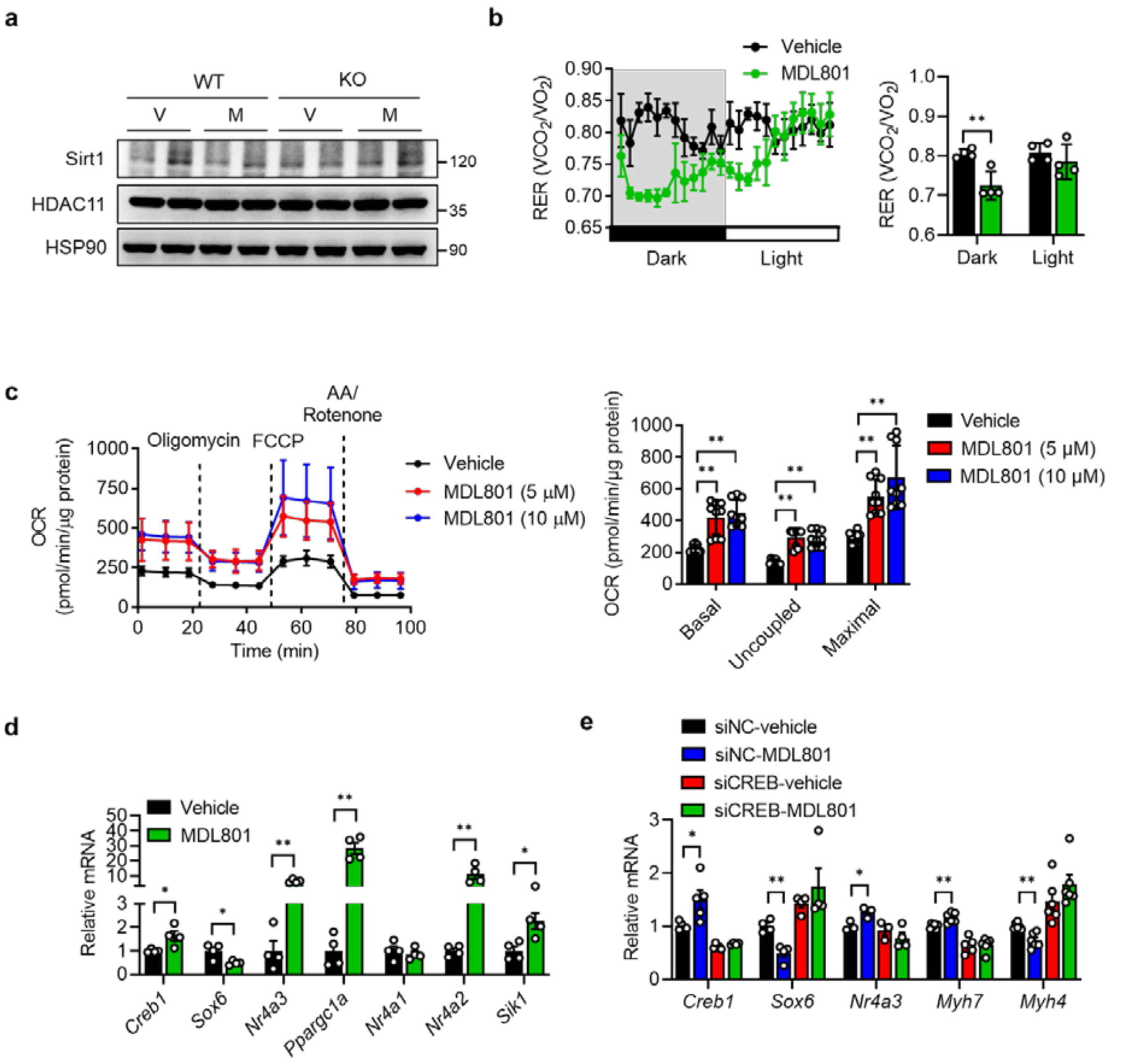

Supplementary Figure 16. Increase in endurance exercise performance in mice treated with Sirt6 activator. a Protein expression of Sirt1 and HDAC11 was examined in gastrocnemius muscles of WT and Sirt6 KO mice treated with MDL801. **b** The respiratory exchange ratio (RER) was compared between MDL801- and vehicle-treated mice (n=4). Twenty-five-week-old mice were treated every day with MDL801 (100 mg/kg) via oral gavage for four weeks during treadmill exercise program. **c** Oxygen consumption rate (OCR) was measured in C2C12 treated with MDL801 (10 µM) for 16 h (n=9). **d** qPCR analysis of *Creb1* and its target genes in gastrocnemius muscles of WT mice (n=4). **e** Expression of *Sox6* and myofiber specific genes was measured by qPCR in C2C12 myotubes treated with MDL801 (10 µM) after transfection with negative control (siNC) or CREB siRNA (n=5). Values are mean ± SEM. All Western blot images and data are representative of at least three independent experiments. Unpaired two-tailed *t*-test between two groups (**b, d**) and one-way ANOVA followed by Bonferroni's *post hoc* analysis (**c, e**) were conducted for statistical analyses. *, p<0.05 and **, p<0.01. V, vehicle; M, MDL801. Source data are provided as a Source Data file.

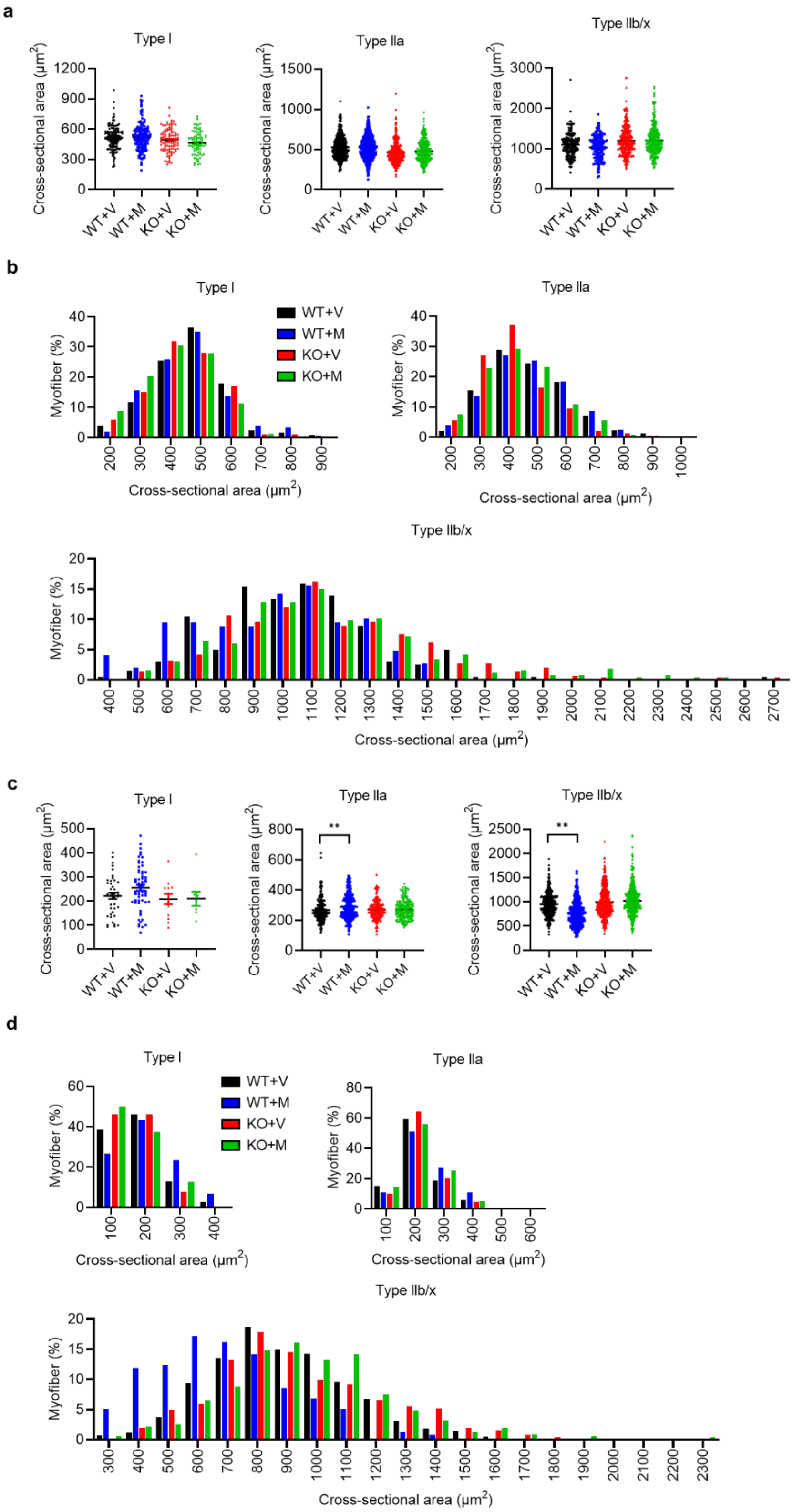

Supplementary Figure 17. Myofiber size distribution histograms in gastrocnemius (a, b) and extensor digitorum longus muscles (c, d) of MDL801-treated mice. On the basis of expression of MyHC-positive myofibers in Figure 6e, the cross-sectional area of each type was determined (n=5). Data are representative of at least three independent experiments (**a**, **c**). One-way ANOVA followed by Bonferroni's *post hoc* analysis was conducted for statistical analyses. **, p<0.01. V, vehicle; M, MDL801. Source data are provided as a Source Data file.

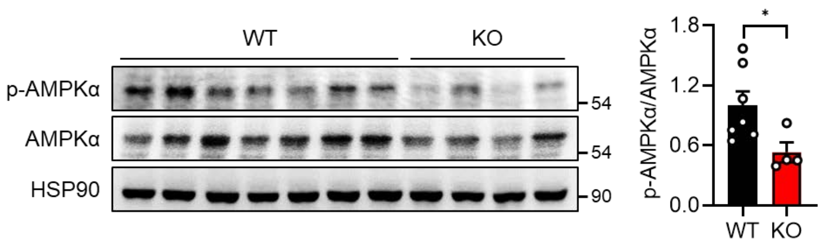

Supplementary Figure S18. Reduction of AMPK activity in *Sirt6* KO mice at basal condition. The levels of total- and phosphorylated AMPKα were examined by Western blotting in gastrocnemius muscles of twenty-five-week-old WT or Sirt6 KO mice (n=7 for WT, n=4 for KO). Values are mean ± SEM. All Western blot images and data are representative of at least three independent experiments. Unpaired two-tailed *t*-test between two groups was conducted for statistical analyses. *, $p<0.05$. Source data are provided as a Source Data file.
