# Supplementary material for: Sirt6 reprograms myofibers to oxidative type through CREB-dependent Sox6 suppression
Source: Nat Commun. 2022 Apr 4;13:1808. doi: 10.1038/s41467-022-29472-5 (PMC8980083; doi:10.1038/s41467-022-29472-5)
Supplement: Supplementary file 2 — Reporting Summary [file 41467_2022_29472_MOESM2_ESM.pdf]

nature portfolio

Corresponding author(s): Byung-Hyun Park, MD-PhD
Eun Ju Bae, PhD

Last updated by author(s): March 8, 2022

Reporting Summary

Nature Portfolio wishes to improve the reproducibility of the work that we publish. This form provides structure for consistency and transparency in reporting. For further information on Nature Portfolio policies, see our Editorial Policies and the Editorial Policy Checklist.

Statistics

For all statistical analyses, confirm that the following items are present in the figure legend, table legend, main text, or Methods section.

n/a	Confirmed	
☐	☒	The exact sample size (n) for each experimental group/condition, given as a discrete number and unit of measurement
☐	☒	A statement on whether measurements were taken from distinct samples or whether the same sample was measured repeatedly
☐	☒	The statistical test(s) used AND whether they are one- or two-sided<br>*Only common tests should be described solely by name; describe more complex techniques in the Methods section.*
☐	☒	A description of all covariates tested
☐	☒	A description of any assumptions or corrections, such as tests of normality and adjustment for multiple comparisons
☒	☐	A full description of the statistical parameters including central tendency (e.g. means) or other basic estimates (e.g. regression coefficient) AND variation (e.g. standard deviation) or associated estimates of uncertainty (e.g. confidence intervals)
☐	☒	For null hypothesis testing, the test statistic (e.g. F, t, r) with confidence intervals, effect sizes, degrees of freedom and P value noted<br>*Give P values as exact values whenever suitable.*
☒	☐	For Bayesian analysis, information on the choice of priors and Markov chain Monte Carlo settings
☒	☐	For hierarchical and complex designs, identification of the appropriate level for tests and full reporting of outcomes
☐	☒	Estimates of effect sizes (e.g. Cohen's d, Pearson's r), indicating how they were calculated

Our web collection on statistics for biologists contains articles on many of the points above.

Software and code

Policy information about availability of computer code

Data collection	RNA Seq was done by the Illumina Hiseq 4000 system. Microscopic imaging was done by Hitachi Bio-TEM and Leica DM750 microscope.
Data analysis	RNA-seq: STAR RNA-weq aligner sorfware (version STAR_2.5.0a)<br>Gene Set Enrichment Analysis (GSEA, Broad Institute, version 3.0)<br>Isometric force and fatigue measurement: LabChart Pro Software (version 8)<br>Image analysis: iSolution DT 36 software (version 11)<br>Western blot analysis: Las-4000 imager (version 1.2)<br>Plotting and Statistics: SPSS (version 27) and Graphpad Prism (version 9.3)

For manuscripts utilizing custom algorithms or software that are central to the research but not yet described in published literature, software must be made available to editors and reviewers. We strongly encourage code deposition in a community repository (e.g. GitHub). See the Nature Portfolio guidelines for submitting code & software for further information.

Data

Policy information about availability of data

All manuscripts must include a data availability statement. This statement should provide the following information, where applicable:

- Accession codes, unique identifiers, or web links for publicly available datasets
- A description of any restrictions on data availability
- For clinical datasets or third party data, please ensure that the statement adheres to our policy

The source data underlying all box plots, bar, and line graphs can be found in the online Source Data File as well as the original uncropped Western blots. Raw and processed RNA-seq datasets were deposited to NCBI's GEO database under the accession number (GSE186105). RNA-seq data can be accessed using the link

https://www.ncbi.nlm.nih.gov/geo/query/acc.cgi?acc=GSE186105. Public microarray data (GSE9103) can be accessed using the link https://www.ncbi.nlm.nih.gov/geo/query/acc.cgi?acc=GSE9103.

Field-specific reporting

Please select the one below that is the best fit for your research. If you are not sure, read the appropriate sections before making your selection.

[x] Life sciences [ ] Behavioural & social sciences [ ] Ecological, evolutionary & environmental sciences

For a reference copy of the document with all sections, see nature.com/documents/nr-reporting-summary-flat.pdf

Life sciences study design

All studies must disclose on these points even when the disclosure is negative.

Sample size	For in vitro and in vivo experiments, sample size selection was based on literature (PMID: 28263310, 31519904, 21109195, and 26299309) and the lab's previous experience. We usually observed standard deviation of around 20% and therefore average of 6 mice per group is sufficient to detect 33% change with 80% power. For signaling assays, we typically performed at least three independent experiment repeats to allow statistical analysis and robust conclusions to be drawn. For RNA-seq analysis, the sample size was determined by minimum requirement for statistical analysis (three samples).
Data exclusions	No data exclusions in this manuscript.
Replication	For all experiments we used multiple biological replicates, as indicated in the figure legends. For quantitative measurements, three or more independent experiments were carried out and statistical analysis performed. For non-quantitative experiments, each experimental condition was repeated at least one more time to confirm the observation.
Randomization	Mice were randomized to treatment group from age matched littermates. For all in vitro studies, randomization is not relevant because all the cells were under identical conditions before different treatment samples applied.
Blinding	Investigators were blinded to group allocation during data collection and data analyses.

Reporting for specific materials, systems and methods

We require information from authors about some types of materials, experimental systems and methods used in many studies. Here, indicate whether each material, system or method listed is relevant to your study. If you are not sure if a list item applies to your research, read the appropriate section before selecting a response.

Materials & experimental systems

n/a	Involved in the study	
[ ]	[x]	Antibodies
[ ]	[x]	Eukaryotic cell lines
[x]	[ ]	Palaeontology and archaeology
[ ]	[x]	Animals and other organisms
[x]	[ ]	Human research participants
[x]	[ ]	Clinical data
[x]	[ ]	Dual use research of concern

Methods

n/a	Involved in the study	
[x]	[ ]	ChIP-seq
[x]	[ ]	Flow cytometry
[x]	[ ]	MRI-based neuroimaging

Antibodies

Antibodies used

All antibodies used for Western blotting, immunofluorescence, and chromatin immunoprecipitation analyses are provided as follows:
Western blot
Sirt6 (#12486, 1:1,000 dilution, Cell Signaling Technology)
CREB (#9197, 1:1,000 dilution, Cell Signaling Technology)
p-CREB (#9198 1:1,000 dilution, Cell Signaling Technology)
p-AMPKα (#50081, 1:1,000 dilution, Cell Signaling Technology)
AMPKα (#5831, 1:1,000 dilution, Cell Signaling Technology)
Drp1 (sc-271583, 1:1,000 dilution, Santa Cruz Biochemicals)
Fis1 (sc-376447, 1:1,000 dilution, Santa Cruz Biochemicals)
Nor1 (sc-393902, 1:1,000 dilution, Santa Cruz Biochemicals)
Nur77 (sc-365113, 1:1,000 dilution, Santa Cruz Biochemicals)
Lamin B1 (sc-6216, 1:1,000 dilution, Santa Cruz Biochemicals)
Sirt1 (sc-74504, 1:1,000 dilution, Santa Cruz Biochemicals)
HDAC11 (sc-390737, 1:1,000 dilution, Santa Cruz Biochemicals)
Ac-H3K9 (H9286, 1:1,000 dilution, Sigma-Aldrich)

HSP90 (ADI-SPA-836-F, 1:1,000 dilution, Enzo Life Sciences)
Total OXPHOS antibody cocktail (ab110413, 1:1,000 dilution, Abcam)
Sox6 (ab64946, 1:1,000 dilution, Abcam)
Mfn1 (ab57602, 1:1,000 dilution, Abcam)
OPA1 (612606, 1:1,000 dilution, BD Biosciences)
PGC-1α (AB-3242, 1:1,000 dilution, Millipore)
RNA Pol II (920101, 1:2,500 dilution, BioLegend)

Chromatin immunoprecipitation
Sirt6 (#12486, 1:200 dilution, Cell Signaling Technology)
Sox6 (ab64946, 1:250 dilution, Abcam)
CREB (MA1-083, 1:250 dilution Cell Signaling Technology)
Ac-H3K9 (H9286, 1:250 dilution, Sigma-Aldrich)
RNA Pol II (920101, 1:250 dilution, BioLegend)
IgG (#2729, 1:250 dilution, Cell Signaling Technology)

Immunohistochemistry
α-myosin (M4276, 1:400 dilution, Sigma-Aldrich)
β-galactosidase (A11132, 1:100 dilution, Thermo Fisher Scientific)
MyHC1 (BA-D5, 1:100 dilution, Developmental Studies Hybridoma Bank (DSHB)
MyHC2a (SC-71, 1:100 dilution, Developmental Studies Hybridoma Bank (DSHB)
MyHC2b (BF-F3, 1:50 dilution, Developmental Studies Hybridoma Bank (DSHB)
Alexa Fluor 350-conjugated goat anti-mouse IgG2b (A21140, 1:100 dilution, Thermo Fisher Scientific)
Alexa Fluor 488-conjugated goat anti-mouse IgG1 (A21121, 1:100 dilution, Thermo Fisher Scientific)
Alexa Fluor 594-conjugated goat anti-mouse IgM (A21044, 1:100 dilution, Thermo Fisher Scientific)

Validation

All antibodies used in this study were commercially developed and validated by the companies.
Sirt6, https://www.cellsignal.com/products/primary-antibodies/sirt6-d8d12-rabbit-mab/12486?site-search-type=Products&N=4294956287&Ntt=%2312486&fromPage=plp&_requestid=1398018
CREB, https://www.cellsignal.com/products/primary-antibodies/creb-48h2-rabbit-mab/9197
p-CREB, https://www.cellsignal.com/products/primary-antibodies/phospho-creb-ser133-87g3-rabbit-mab/9198
p-AMPKα, https://www.cellsignal.com/products/primary-antibodies/phospho-ampka-thr172-d4d6d-rabbit-mab/50081
AMPKα, https://www.cellsignal.com/products/primary-antibodies/ampka-d5a2-rabbit-mab/5831
Drp1, https://www.scbt.com/ko/p/drp1-antibody-c-5
Fis1, https://www.scbt.com/ko/p/fis1-antibody-b-5
Nor1, https://www.scbt.com/ko/p/nor-1-antibody-h-7
Nur77, https://www.scbt.com/ko/p/nur77-antibody-c-5
Lamin B1, https://www.scbt.com/ko/p/lamin-b-antibody-c-20
Sirt1, https://www.scbt.com/ko/p/sirt1-antibody-b-10
HDAC11, https://www.scbt.com/ko/p/hdac11-antibody-c-5
Ac-H3K9, https://www.sigmaaldrich.com/KR/ko/product/sigma/h9286
HSP90, https://www.enzolifesciences.com/ADI-SPA-836/hsp90-polyclonal-antibody/
Total OXPHOS antibody cocktail, https://www.abcam.com/total-oxphos-rodent-wb-antibody-cocktail-ab110413.html
Sox6, https://www.abcam.com/sox6-antibody-ab64946.html
Mfn1, https://www.abcam.com/mitofusin-2--mitofusin-1-antibody-3c9-ab57602.html
OPA1, https://www.labome.com/product/BD-Biosciences/612606.html
PGC-1α, https://www.merckmillipore.com/KR/ko/product/Anti-PGC-1-Antibody,MM_NF-AB3242
RNA Pol II, https://www.biolegend.com/en-us/search-results/purified-anti-rna-polymerase-ii-rpb1-antibody-11666
IgG, https://www.cellsignal.com/products/primary-antibodies/normal-rabbit-igg/2729
α-myosin, https://www.sigmaaldrich.com/KR/ko/product/sigma/m4276
β-galactosidase, https://www.thermofisher.com/antibody/product/beta-Galactosidase-Antibody-Polyclonal/A-11132
MyHC1, https://dshb.biology.uiowa.edu/BA-D5
MyHC2a, https://dshb.biology.uiowa.edu/SC-71
MyHC2b. https://dshb.biology.uiowa.edu/BF-F3
Alexa Fluor 350-conjugated goat anti-mouse IgG2b, https://www.thermofisher.com/antibody/product/Goat-anti-Mouse-IgG2b-Cross-Adsorbed-Secondary-Antibody-Polyclonal/A-21140
Alexa Fluor 488-conjugated goat anti-mouse IgG1, https://www.thermofisher.com/antibody/product/Goat-anti-Mouse-IgG1-Cross-Adsorbed-Secondary-Antibody-Polyclonal/A-21121
Alexa Fluor 594-conjugated goat anti-mouse IgM, https://www.thermofisher.com/antibody/product/Goat-anti-Mouse-IgM-Heavy-chain-Cross-Adsorbed-Secondary-Antibody-Polyclonal/A-21044

Eukaryotic cell lines

Policy information about cell lines

Cell line source(s)

C2C12 cells (CRL-1722) and HEK293T cells (CRL-3216) were purchased from ATCC.

Authentication

Cell lines were authenticated by ourselves. The isolation and authentication of myofibers have been reported in our previous manuscript (10.1002/jcsm.12774).

Mycoplasma contamination	All cells tested negative for mycoplasma.
Commonly misidentified lines (See ICLAC register)	No commonly misidentified cell lines were used in the study.

Animals and other organisms

Policy information about studies involving animals; ARRIVE guidelines recommended for reporting animal research

Laboratory animals	Sirt6flox/flox mice (B6;129-Sirt6tm1Ygu/J, Stock No: 008041), Myl1/MLC1f-Cre mice (Myl1tm1(cre)sjb/J, Stock No: 024713), Sirt6 Tg mice (C57BL/6-Tg(RP23-352G18)1Coppa/J, Stock No: 028361), and R26R reporter mice (FVB.129S4(B6)-Gt(ROSA)26Sortm1Sor/J, Stock No: 009427) were obtained from the Jackson Laboratory (Bar Harbor, ME, USA). Studies were performed in male aged-matched mice between 15 weeks to 25 weeks of age.
Wild animals	No wild animals were used in the study.
Field-collected samples	No filed-collected samples were used in the study.
Ethics oversight	All animal experiments were performed in accordance with the Guide for the Care and Use of Laboratory Animals published by the US National Institutes of Health (NIH Publication No. 85-23, revised 2011). The study protocol was approved by the Institutional Animal Care and Use Committee of Chonbuk National University (permit number: CBNU 2017-0087).

Note that full information on the approval of the study protocol must also be provided in the manuscript.
